# Neurofilaments in CSF As Diagnostic Biomarkers in Motor Neuron Disease: A Meta-Analysis

**DOI:** 10.3389/fnagi.2016.00290

**Published:** 2016-11-29

**Authors:** Dawei Li, Dongchao Shen, Hongfei Tai, Liying Cui

**Affiliations:** ^1^Department of Neurology, Peking Union Medical College Hospital, Chinese Academy of Medical Sciences and Peking Union Medical CollegeBeijing, China; ^2^Neuroscience Center, Chinese Academy of Medical SciencesBeijing, China

**Keywords:** motor neuron disease, amyotrophic lateral sclerosis, CSF biomarkers, neurofilaments, diagnostic value, meta-analysis

## Abstract

**Objective:** Neurofilaments in CSF are promising biomarkers which might help in the diagnosis of motor neuron disease (MND). We aim to assess the diagnostic value of neurofilaments in CSF for MND.

**Methods:** Pubmed, Emabase, and Web of Science were searched for relevant studies systematically. Articles in English that evaluated the utility of neurofilaments in CSF in the diagnosis of MND were included. Data were extracted by two independent investigators. Diagnostic indexes for neurofilament light chain (NFL) and phosphorylated neurofilament heavy chain (pNFH) were calculated separately. Stata 12.0 software with a bivariate mixed-effects model was used to summarize the diagnostic indexes from eligible studies.

**Results:** Five studies on NFL and eight studies on pNFH met inclusion criteria. For NFL, the pooled sensitivity and specificity were 81% (95% confidence interval [CI], 72–88%) and 85% (95% CI, 76–91%), respectively; the positive likelihood ratio (PLR) and negative likelihood ratio (NLR) were 5.5 (95% CI, 3.1–9.8) and 0.22 (95% CI, 0.14–0.35), respectively; the summary diagnostic odds ratio (DOR) was 25 (95% CI, 9–70), and the area under summary receiver operator characteristic curve (AUC) was 0.90 (95% CI, 0.87–0.92). For pNFH, the pooled sensitivity, specificity, PLR and NLR were 85% (95% CI, 80–88%), 85% (95% CI, 77–90%), 5.5 (95% CI, 3.6–8.4), and 0.18 (95% CI, 0.13–0.25), respectively; the DOR was 30 (95% CI, 16–58), and the AUC was 0.91 (95% CI, 0.88–0.93).

**Conclusion:** Neurofilaments in CSF have a high value in the diagnosis of MND, though the optimal cutoff value remains to be further investigated.

## Introduction

Motor neuron diseases (MND) are a group of progressive neurodegenerative disorders characterized by motor neuron loss in the motor cortex, brainstem, and spinal cord. The most common form is amyotrophic lateral sclerosis (ALS), which affects both upper motor neuron (UMN) and lower motor neuron (LMN). Early diagnosis of ALS remains to be a challenge worldwide. Population studies have shown that the average diagnosis latency is about 12 months (Zoccolella et al., 2006), and patients who fulfill the revised El Escorial criteria for clinically definite ALS are usually in the advanced stage of the disease (Turner and Talbot, 2013). Validated biomarkers that can facilitate earlier diagnosis of ALS are urgently needed in order to enable disease-modifying drugs to be administered at an earlier stage.

Neurofilaments are the most abundant neuronal cytoskeletal proteins and are essential to the structural integrity of neurons. Neurofilament subunits, mainly neurofilament light chain (NFL) and phosphorylated neurofilament heavy chain (pNFH), are actively involved in the pathogenesis of axonal injury and degeneration both as causative agents and progression markers for neurological diseases (Petzold, 2005). ALS is characterized by loss of large axons with abundant neurofilaments, and perikaryal accumulation of phosphorylated neurofilaments has been found to occur in ALS (Manetto et al., 1988). Several studies and meta-analysis have showed that concentrations of NFL and pNFH in CSF are significantly increased in patients with ALS (Xu et al., 2016), suggesting that they might be promising neurochemical diagnostic biomarkers for ALS. To fully understand the diagnostic performance of NFL and pNFH for MND, we performed the present meta-analysis to summarize their diagnostic indexes.

## Materials and Methods

### Inclusion of Studies

Pubmed, Embase databases and Web of Science were searched for studies published up to October 31st, 2016 that reported neurofilament concentrations in CSF in patients with MND. Search terms included (‘motor neuron disease’ or ‘MND’ or ‘amyotrophic lateral sclerosis’ or ‘ALS’) AND (‘NFL’ or ‘NEFL’ or ‘NFH’ or ‘NEFH’ or ‘pNFH’ or ‘neurofilament’ or ‘neurofilaments’ or ‘light chain’ or ‘heavy chain’) AND (‘cerebrospinal fluid’ or ‘cerebrospinal fluids’ or ‘CSF’ or ‘biomarker’ or ‘biomarkers’ or ‘biological marker’ or ‘biological markers’). Both text word and MeSH subject headings were used. Language was confined to English, and publication type of review, case reports and letter was excluded in the advanced search. The search strategy was supplemented by inspecting the reference lists of included articles. The studies were considered for inclusion if they (1) evaluated the utility of neurofilament concentrations in CSF for the diagnosis of MND; (2) enrolled healthy controls or patients with neurological disorders other than MND as controls; (3) provide enough data to construct a 2 × 2 table for the diagnostic accuracy. Studies were excluded if they were in line with the following criteria: (1) there was no control group; (2) measured neurofilament concentrations in biological samples other than CSF, including plasma, serum, spinal cords, or brain tissue from biopsy; (3) used non-quantitative methods such as western blot, or assessed the diagnostic accuracy of anti-neurofilament antibodies for MND; (4) had overlapped sample or the sample size <10; (5) could not provide valid data after contacting the authors.

### Data Extraction and Quality Assessment

For each included study, the following data were extracted by two investigators independently using a standard form: country of origin, number of centers, number of cases, patient type, ALS diagnostic criteria, control type, study design, testing method, mean or median value of neurofilaments, cutoff value, and diagnostic indexes. The Quality Assessment of Diagnostic Accuracy Studies-2 (QUADAS-2) tool was used to assess the quality of included studies and their risk of bias (Whiting et al., 2011). The figure of risk of bias and applicability concerns summary was produced using Revman 5.3 software. Any conflicts were resolved by a third party after discussion of each item.

### Data Analysis

We used STATA software (version 12.0, Stata Corporation, 93 College Station, TX, USA) to perform the meta-analysis. A bivariate mixed-effects model was used to analyze the estimates of sensitivity, specificity, positive likelihood ratio (PLR), negative likelihood ratio (NLR), and diagnostic odds ratio (DOR) with 95% confidence interval (CI). The summary receiver operator characteristic (SROC) curve was constructed and the area under the SROC curve (AUC) was calculated to evaluate the overall performance of CSF neurofilaments in MND patients. *P* < 0.1 for *Q*-test or *I*^2^ ≥ 50% for *I*^2^ statistics indicated substantial heterogeneity, in which case heterogeneity test would be performed. The leave-one-out sensitivity analyses were conducted to test the replicability of the results, which consists of repeating the main analysis by removing each study one at a time to recalculate the stability of the remaining studies. Subgroup analyses for pNFH according to study design, testing method, cutoff value, patient type, diagnosis criteria, control type, and population were also carried out. Deeks’ funnel plots were used to test for the potential presence of publication bias, and *P* < 0.05 was considered statistically significant.

## Results

### Search Results and Characteristics of Included Studies

**FIGURE 1:**
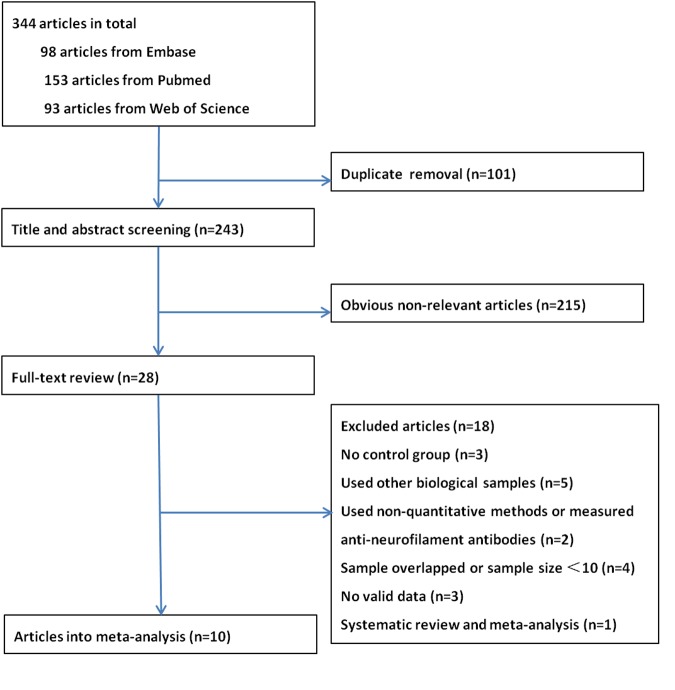
**The flow chart of the literature search in the meta-analysis**.

A total of 344 articles were identified. After removal of duplicate entries, 243 articles remained and then were screened by title and abstract. As a result, 28 full-text articles were assessed for eligibility, of which three had no control group (Boylan et al., 2013; Tortelli et al., 2015; Weydt et al., 2016), five investigated neurofilament levels in other biological samples (Troost et al., 1992; Strong et al., 2001; Mendonca et al., 2005; Puentes et al., 2014; McCombe et al., 2015), one utilized western blot (Mendonca et al., 2011) and one measured anti-neurofilament antibodies (Fialova et al., 2010), two had overlapping data sets (Brettschneider et al., 2006; Goncalves et al., 2015) and two had a sample size <10 (Norgren et al., 2003; Petzold et al., 2003), and three did not provide sufficient data to allow construct a 2 × 2 table (Kuhle et al., 2010; Gaiottino et al., 2013; Lehnert et al., 2014), one was a systematic review and meta-analysis (Xu et al., 2016). Finally, 10 articles were included in the meta-analysis, of which two reported data on NFL only (Tortelli et al., 2012; Lu et al., 2015), five reported on pNFH only (Ganesalingam et al., 2011, 2013; Chen et al., 2016; Goncalves et al., 2016; Li et al., 2016) and three reported both (Reijn et al., 2009; Steinacker et al., 2015; Oeckl et al., 2016). A flow chart of publication selection is presented in **Figure 1**. Of note, one study that focused on multicenter validation of CSF neurofilaments as diagnostic biomarkers for ALS enrolled participants from 15 centers across Europe and America (Oeckl et al., 2016), part of which might overlapped with other four studies (Ganesalingam et al., 2013; Lu et al., 2015; Steinacker et al., 2015; Goncalves et al., 2016). However, the multicenter study only recruited five ALS patients and five controls from each center; therefore, we reckoned that the multiple publication bias, if existed, could be ignored in view of the relative large total sample size. The basic characteristics of each study are shown in **Table 1**. More details please refer to the **Supplementary Data Sheet 1**.

**Table 1:** Basic characteristics of included studies.

Study	Country	Center (s)	Testing target (cutoff, pg/mL)	Patients/Controls	Patient type	Diagnostic criteria	Control type	Study design	Testing method	SEN	SPE
Li et al., 2016	China	1	pNFH (589)	51/42	ALS	R- El Escorial	NC and HC	Case-control	ELISA (BioVendor)	82.4%	73.8%
Chen et al., 2016	China	1	pNFH (437)	40/40	ALS	R- El Escorial	NC	Cross-sectional	ELISA (BioVendor)	97.3%	83.8%
Oeckl et al., 2016	Europe and America	15	NFL (1431)	57/66	ALS	R- El Escorial	NC and HC	Case-control	ELISA (IBL)	79.0%	86.4%


			pNFH (568.5)	75/75					ELISA (BioVendor)	78.7%	93.3%
Goncalves et al., 2016	Portugal	1	pNFH (554)	36/15	ALS	R- El Escorial	NC	Case-control	ELISA (BioVendor)	75%	60%
Lu et al., 2015	UK	1	NFL (1781)	38/20	ALS	R- El Escorial	HC	Cross-sectional	ECL- immunoassay	97%	95%
Steinacker et al., 2015	German	1	NFL (2200)	253/202	MND	R- El Escorial	NC	Case-control	ELISA (IBL)	79%	85%


			pNFH (560)						ELISA (Biovendor)	84%	77%
Ganesalingam et al., 2013	Sweden	1	pNFH (1200)	150/140	ALS	Unknown	NC	Case-control	ELISA (BioVendor)	90%	87%
Tortelli et al., 2012	Italy	1	NFL (1981)	37/46	ALS	El Escorial	NC	Cross-sectional	ELISA (Norgren et al., 2003)	78.4%	72.5%
Ganesalingam et al., 2011	USA	2	pNFH (635)	71/92	ALS	El Escorial	NC and HC	Case-control	ELISA (BioVendor)	87.7%	93.7%
Reijn et al., 2009	Netherland	1	NFL (22.6)	28/14	ALS	R- El Escorial	NC	Case-control	ELISA (Van Geel et al., 2005)	75%	79%
			pNFH (502)	29/15					SMI35 pNfH Ab (Stenberger)	72%	80%

*ALS, amyotrophic lateral sclerosis; ECL, electrochemiluminescence; HC, healthy control; MND, motor neuron disease; NC, neurological control; NFL, neurofilament light chain; pNFH, phosphorylated neurofilament heavy chain; R- El Escorial, revised El Escorial; SEN, sensitivity; SPE, specificity.*

### Quality Assessment

**FIGURE 2:**
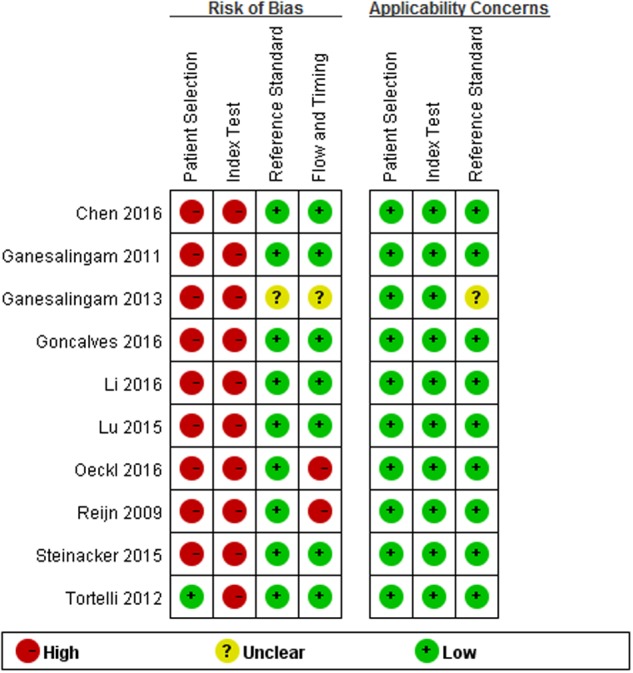
**Methodological quality summary**.

Quality assessment results based on QUADAS-2 are shown in **Figure 2**. Concerning the domain of patient selection, only three studies were cross-sectional (Tortelli et al., 2012; Lu et al., 2015; Chen et al., 2016), while other seven studies were case-control designed; only one study reported that their patients were enrolled consecutively, and two studies reported their controls were age- and sex-matched to cases (Tortelli et al., 2012; Chen et al., 2016), and two studies reported the controls were only age-matched to the cases (Ganesalingam et al., 2011; Li et al., 2016), while other studies did not describe these information explicitly; four studies used healthy controls who “came from the community,” “were typically spouses and friends of patients,” “were initially presented with neurological symptoms and underwent lumber puncture as a part of the diagnostic examinations” but turned out to be without any neurological disease, or were not specified. (Ganesalingam et al., 2011; Lu et al., 2015; Li et al., 2016; Oeckl et al., 2016); neurological controls usually included the following: ALS mimics, other neurodegenerative diseases, inflammatory conditions, and other neurological diseases. More information about the neurological controls in each included study please refer to the **Supplementary Data Sheet 1**. As for the index test, all studies selected the test threshold to optimize sensitivity and specificity instead of using a pre-specified threshold. Two studies had a high risk of the flow and timing item, since some of the patients enrolled in the studies were not included in the final analysis (Reijn et al., 2009; Oeckl et al., 2016). One study did not report explicitly its diagnostic criteria for ALS, therefore its risk of reference standard remains unclear (Ganesalingam et al., 2013).

### Diagnostic Accuracy

**FIGURE 3:**
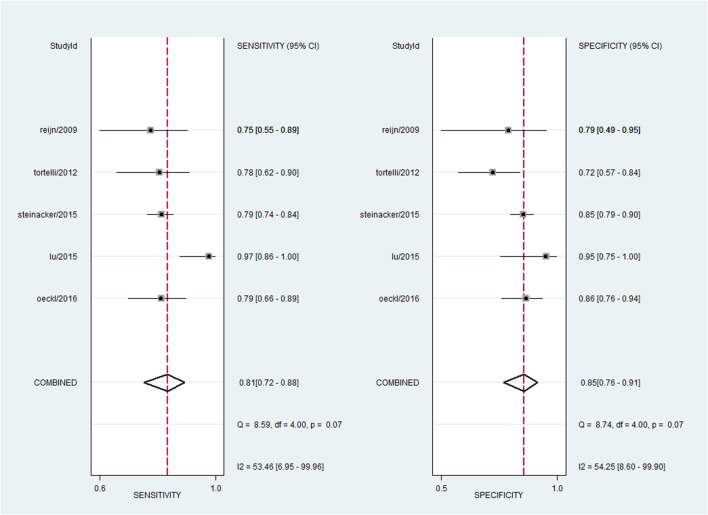
**Forest plots showing the sensitivity and specificity of NFL in the diagnosis of MND**.

**FIGURE 4:**
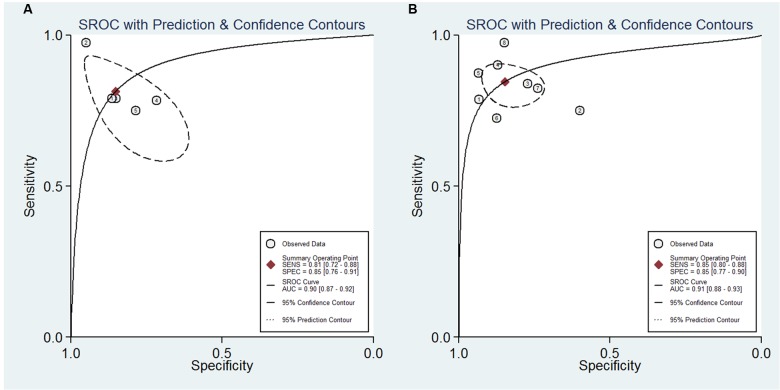
**Summary receiver operator characteristic of the accuracy of NFL (A)** and pNFH **(B)** in the diagnosis of MND.

For NFL, the pooled sensitivity, specificity, PLR and NLR were 81% (95% CI, 72–88%), 85% (95% CI, 76–91%), 5.5 (95% CI, 3.1–9.8), and 0.22 (95% CI, 0.14–0.35), respectively; the summary DOR was 25 (95% CI, 9–70), and the AUC was 0.90 (95% CI, 0.87–0.92) (**Figures 3** and **4A**).

**FIGURE 5:**
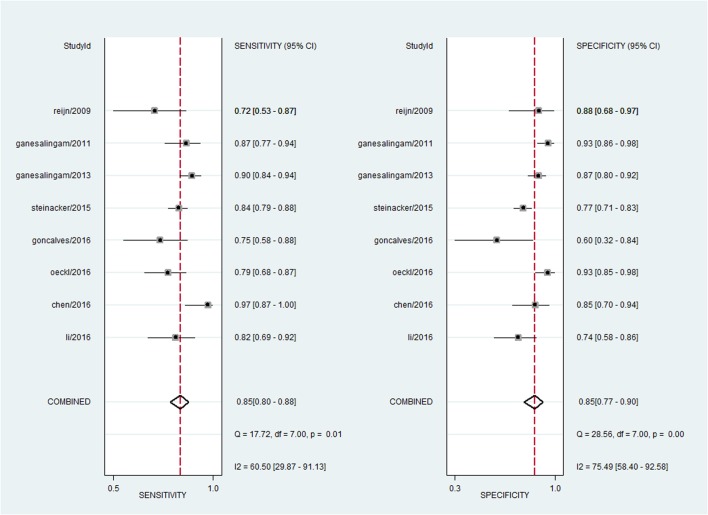
**Forest plots showing the sensitivity and specificity of pNFH in the diagnosis of MND**.

For pNFH, the pooled sensitivity, specificity, PLR and NLR were 85% (95% CI, 80–88%), 85% (95% CI, 77–90%), 5.5 (95% CI, 3.6–8.4), and 0.18 (95% CI, 0.13–0.25), respectively; the summary DOR was 30 (95% CI, 16–58), and the AUC was 0.91 (95% CI, 0.88–0.93) (**Figures 4B** and **5**).

### Exploration of Heterogeneity and Publication Bias

**FIGURE 6:**
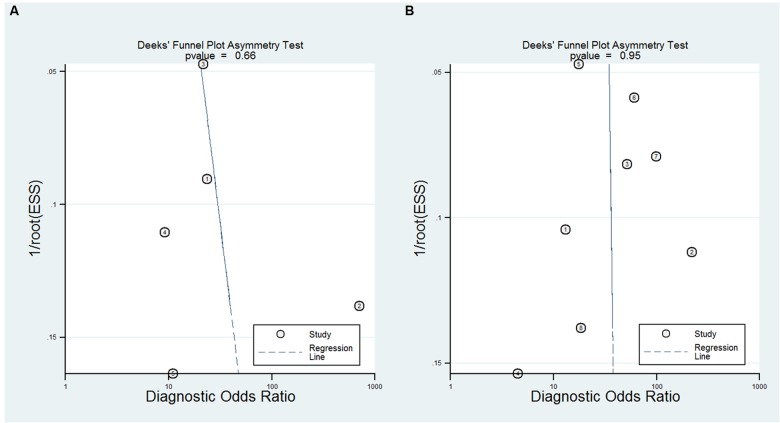
**The Deeks’ test of the diagnostic meta-analysis [(A)** NFL and **(B)** pNFH].

A bivariate mixed-effects model was used in this meta-analysis. No significant heterogeneity was found among studies on NFL (*P* = 0.493, *I*^2^ = 0), and moderate heterogeneity was detected among studies on pNFH (*P* = 0.077, *I*^2^ = 47). Sensitivity analyses indicated that results of the main meta-analysis were stable for NFL (**Table 2**). However, in the leave-one-out analysis for pNFH, the moderate heterogeneity disappeared when Ganesalingam et al. (2011; *I*^2^= 0) or Oeckl et al. (2016; *I*^2^ = 8) was removed, suggesting they might be the sources of heterogeneity. In the subgroup analyses for pNFH, when studies were confined to those used neurological controls only, the heterogeneity also disappeared (**Table 3**). The results of Deeks’ test did not reveal significant publication bias in this study (**Figure 6**).

**Table 2:** Results of sensitivity analyses.

Variables	*P* for *Q*-test	*I*^2^ statistics	Sensitivity (95% CI)	Specificity (95% CI)	PLR (95% CI)	NLR (95% CI)	DOR (95% CI)	AUC (95% CI)
**Sensitivity analysis for NFL**								
Oeckl et al., 2016	0.496	0	0.82 (0.69–0.91)	0.86 (0.73–0.93)	5.7 (2.6–12.7)	0.21 (0.10–0.41)	28 (6–119)	0.91 (0.88–0.93)
Lu et al., 2015	0.492	0	0.79 (0.74–0.83)	0.83 (0.76–0.88)	4.6 (3.2–6.5)	0.26 (0.21–0.32)	18 (11–30)	0.83 (0.79–0.86)
Steinacker et al., 2015	0.489	0	0.83 (0.71–0.91)	0.84 (0.71–0.92)	5.3 (2.5–11.4)	0.20 (0.10–0.39)	26 (7–101)	0.90 (0.88–0.93)
Tortelli et al., 2012	0.426	0	0.84 (0.71–0.91)	0.87 (0.80–0.92)	6.5 (3.7–11.5)	0.19 (0.10–0.36)	35 (11–109)	0.92 (0.89–0.94)
Reijn et al., 2009	0.497	0	0.83 (0.71–0.90)	0.87 (0.76–0.93)	6.2 (3.0–12.9)	0.20 (0.10–0.37)	32 (8–118)	0.92 (0.89–0.94)
**Sensitivity analysis for pNFH**								
Li et al., 2016	0.065	51	0.85 (0.79–0.89)	0.86 (0.79–0.91)	6.1 (3.8–9.6)	0.18 (0.12–0.25)	35 (17–70)	0.92 (0.89–0.94)
Chen et al., 2016	0.041	60	0.83 (0.79–0.87)	0.85 (0.76–0.90)	5.4 (3.3–8.7)	0.20 (0.15–0.26)	28 (14–55)	0.89 (0.86–0.91)
Oeckl et al., 2016	0.361	0	0.86 (0.81–0.89)	0.82 (0.75–0.88)	4.9 (3.2–7.5)	0.17 (0.12–0.25)	28 (13–60)	0.91 (0.88–0.93)
Goncalves et al., 2016	0.064	51	0.85 (0.81–0.89)	0.86 (0.80–0.91)	6.3 (4.2–9.4)	0.17 (0.12–0.23)	37 (21–67)	0.92 (0.89–0.94)
Steinacker et al., 2015	0.112	33	0.85 (0.78–0.90)	0.86 (0.78–0.91)	6.1 (3.7–9.8)	0.18 (0.12–0.26)	34 (16–73)	0.92 (0.89–0.94)
Ganesalingam et al., 2013	0.032	64	0.83 (0.79–0.87)	0.84 (0.75–0.90)	5.3 (3.3–8.7)	0.20 (0.15–0.26)	27 (14–53)	0.88 (0.85–0.91)
Ganesalingam et al., 2011	0.169	8	0.84 (0.78–0.89)	0.83 (0.75–0.88)	4.9 (3.32–7.2)	0.19 (0.13–0.27)	26 (13–49)	0.90 (0.87–0.93)
Reijn et al., 2009	0.091	41	0.85 (0.81–0.89)	0.84 (0.76–0.90)	5.5 (3.4–8.7)	0.17 (0.13–0.24)	32 (16–65)	0.91 (0.88–0.93)

*AUC, area under curve; DOR, diagnostic odd ratio; NFL, neurofilament light chain;NLR, negative likelihood ratio; PLR, positive likelihood ratio; pNFH, phosphorylated neurofilament heavy chain.*

**Table 3:** Results of subgroup analyses for pNFH.

Variables	*P* for *Q*-test	*I*^2^ statistics	Sensitivity (95% CI)	Specificity (95% CI)	PLR (95% CI)	NLR (95% CI)	DOR (95% CI)	AUC (95% CI)
Case-control studies	0.041	60	0.83 (0.79–0.87)	0.85 (0.76–0.90)	5.4 (3.3–8.7)	0.20 (0.15–0.26)	28 (14–55)	0.89 (0.86–0.91)
Cutoff <1000 pg/mL	0.032	64	0.83 (0.79–0.87)	0.84 (0.75–0.90)	5.3 (3.3–8.7)	0.20 (0.15–0.26)	27 (14–53)	0.88 (0.85–0.91)
ELISA method	0.091	41	0.85 (0.81–0.89)	0.84 (0.76–0.90)	5.5 (3.4–8.7)	0.17 (0.13–0.24)	32 (16–65)	0.91 (0.88–0.93)
ALS only	0.112	33	0.85 (0.78–0.90)	0.86 (0.78–0.91)	6.1 (3.7–9.8)	0.18 (0.12–0.26)	34 (16–73)	0.92 (0.89–0.94)
R-El Escorial only	0.08	45	0.82 (0.79–0.86)	0.82 (0.74–0.89)	4.6 (4.0–7.0)	0.21 (0.17–0.26)	22 (13–36)	0.85 (0.82–0.88)
Europe and America	0.04	60	0.83 (0.78–0.88)	0.86 (0.77–0.92)	6.0 (3.5–10.2)	0.19 (0.14–0.26)	31 (15–66)	0.90 (0.87–0.92)
NC only	0.465	0	0.86 (0.78–0.91)	0.81 (0.73–0.87)	4.5 (3.0–6.7)	0.18 (0.11–0.29)	25 (10–62)	0.90 (0.87–0.92)

*ALS, amyotrophic lateral sclerosis; AUC, area under curve; DOR, diagnostic odd ratio; NC, neurological control; NLR, negative likelihood ratio; PLR, positive likelihood ratio; pNFH, phosphorylated neurofilament heavy chain, R-El Escorial, revised El Escorial.*

## Discussion

Neurofilament proteins are essential for maintaining normal axonal caliber and conduction velocity. Elevated levels of NFL and pNFH are reflections of ongoing destruction of axons, as have been shown in MND and other neurodegenerative diseases (Brettschneider et al., 2006). Our study provides a comprehensive meta-analysis of the CSF neurofilaments as diagnostic biomarkers in MND. A high AUC for both biomarkers qualified them as discriminative markers for suspected MND, and the results of sensitivity analyses demonstrated the overall results were stable. However, negative results for these markers cannot rule out MND diagnosis.

Though CSF NFL and pNFH concentrations have been shown to be consistently increased in ALS patients, the cutoff value for diagnosis among studies varies considerably, and the pNFH measurement appears to be more robust than the NFL assay (Li et al., 2016). For pNFH, its concentrations were comparable among different centers and the inter-laboratory variation of measurements was acceptable, partly because it seems to be stable against preanalytical variables and is less affected by blood contamination, repeated freeze-thaw cycles, delayed processing, or storage temperature (Koel-Simmelink et al., 2014). On the contrary, stability remains to be a particular problem for NFL (Koel-Simmelink et al., 2011), which limits its practicability and challenges it as a biomarker to be used in clinical trials. Concerning the assay methods, besides the purchasable ELISA methods, two studies (Reijn et al., 2009; Tortelli et al., 2012) utilized independent specific enzyme immunoassay for the detection of NFL (Norgren et al., 2003; Van Geel et al., 2005), and one study used electrochemiluminescence immunoassay (Lu et al., 2015). Beyond the preanalytical and analytical aspects as reasons for neurofilament cutoff variability among centers, effects of clinical differences also played an important role, especially disease duration, rate of progress and the neurological controls with a variety of syndromes. Studies have shown that CSF pNFH levels are inversely correlated with disease duration (Ganesalingam et al., 2013), while NFL levels changed only minimally throughout most of the disease course in ALS (Lu et al., 2015); CSF pNFH levels were higher in patients with predominantly UMN involvement (Brettschneider et al., 2006), while NFL levels did not differ between patients with predominantly UMN involvement and patients with predominantly LMN involvement (Tortelli et al., 2012); ALS-fast tend to have higher CSF NFL and pNFH levels than ALS-slow (Tortelli et al., 2012; Goncalves et al., 2015; Lu et al., 2015; Chen et al., 2016).

The broad range of neurofilament values might accentuate its general applicability in MND diagnosis. Thus, standardization of the above-mentioned factors in combination with personnel training and regular quality control are now recommended and commonly accepted in order to give reproducible results in different laboratories and to successfully implement neurofilaments determination as diagnostic criteria for ALS (Lehnert et al., 2014; Oeckl et al., 2016). In addition, neurofilament measurements should be ideally undertaken as close as to reported disease onset, when ALS is suspected or at diagnosis.

An important concern for neurofilaments as diagnostic biomarkers is the timing of their increase. Weydt et al. (2016) found that asymptomatic individuals carrying an ALS mutation did not show any trend toward increased levels of NFL and pNFH, even when they were close to the assumed disease onset. However, there was an increase in CSF neurofilament levels between asymptomatic carriers and symptomatic ALS patients and this increase might turn out to be a rather sudden event, possibly reflecting the onset of irreversible structural damage of motor neurons. Their results indicate that neurofilaments are state markers closely related to the symptomatic phase. Longitudinal studies are needed to help determine when the surge in neurofilament levels occurs exactly in relation to the phenotype conversion.

Despite the potential diagnostic accuracy of CSF neurofilaments, serial lumbar punctures for longitudinal neurofilaments monitoring are far less practical than blood sampling, which makes blood-derived neurofilaments level a more favorable surrogate marker for disease staging and prognosis in ALS (Puentes et al., 2014; McCombe et al., 2015). Three studies investigating the correlation between CSF and serum neurofilaments revealed that NFL levels in matched CSF and serum samples were highly correlated (Lu et al., 2015), while the correlation between plasma and CSF levels of pNFH is controversial (Ganesalingam et al., 2011; Li et al., 2016). CSF and blood matrices may act differently on neurofilament homeostasis and clearance depending on its concentrations (Lu et al., 2015). Future studies are required to further explore the relationship between CSF and blood levels of neurofilament and to optimize the detection of neurofilaments in the blood as well.

This meta-analysis had some limitations. First, we only included 10 studies, most of which had a relatively small sample size. Thus, though no significant heterogeneity or publication bias was found, we were unable to conduct meta-regression to further investigate the variability between studies, and to conclude the optimal cutoff value. Second, certain concerns were raised during quality assessment of the included studies. Most studies are case-control designed, which warrants well-designed prospective cohort studies with larger sample size in the future. Furthermore, most studies did not explicitly report whether a consecutive or random sample of patients was enrolled, and four studies used healthy controls, and ROC was used to determine a cutoff to give the optimum sensitivity and specificity in all studies. All of above might lead to overestimation of the test performance. It should also be noted that increased neurofilament level are not specific for ALS and have been reported in a number of other neurological diseases, including multiple sclerosis, dementia and brain trauma (Norgren et al., 2003; Skillback et al., 2014; Arrambide et al., 2016; Kirkcaldie and Collins, 2016; Xu et al., 2016). An ideal study should include other neurodegenerative disorders and ALS mimics as controls and use a pre-specified threshold. Finally, most studies included in the meta-analysis were from western countries, while only two studies were from China and there is no relevant report about other Asian population or African population. This becomes important because ALS patients from Asia are very different and they survive substantially longer than western patients (Nalini et al., 2008; Liu et al., 2014).

## Conclusion

This meta-analysis demonstrated that CSF neurofilaments may currently serve as diagnostic biomarkers for MND. Our data support future prospective studies of neurofilament levels in CSF to determine its clinical utility. Standardized procedures for obtaining and processing CSF, and an improved understanding of how neurofilaments change with the pathology will further strengthen the case for neurofilaments in the diagnosis and therapeutic trials of ALS.

## Author Contributions

DL: literature search, statistical analysis, writing of the first draft. DS: literature search, statistical analysis, writing of the first draft. HT: statistical analysis review and critique. LC: research organization, manuscript review, and critique.

## Conflict of Interest Statement

The authors declare that the research was conducted in the absence of any commercial or financial relationships that could be construed as a potential conflict of interest.

## Supplementary Material

DATA SHEET 1 **More detail information of included studies**.
